# Global Cervical Cancer Incidence by Histological Subtype and Implications for Screening Methods

**DOI:** 10.1007/s44197-023-00172-7

**Published:** 2024-01-03

**Authors:** Minmin Wang, Kepei Huang, Martin C. S. Wong, Junjie Huang, Yinzi Jin, Zhi-Jie Zheng

**Affiliations:** 1 Department of Global Health, School of Public Health, Peking University, 38 Xue Yuan Road, Haidian District, Beijing, 100191 China; 2 Institute for Global Health and Development, Peking University, Beijing, China; 3 The Chinese University of Hong Kong, Shatin, NT Hong Kong SAR, China

**Keywords:** Histological subtype, Adenocarcinoma, Squamous cell carcinoma, Cervical cancer screening

## Abstract

**Background:**

Cervical cancer is a major global health concern, disproportionately affecting women in developing countries. Cervical cancer has two primary subtypes, squamous cell carcinoma (SCC) and adenocarcinoma (AC), each with distinct characteristics and screening effectiveness. In this study, we aimed to estimate the global incidence of cervical cancer according to histological subtype to inform prevention strategies.

**Methods:**

Using data from population-based cancer registries, we computed the rates of SCC, AC, and other specified histology among all cervical cancer cases by country and by 5-year age group. Proportions were subsequently applied to the estimated number of cervical cancer cases from the Global Cancer Observatory 2020. Age-standardized incidence rates were calculated.

**Results:**

SCC accounted for 82.72% of global cervical cancer cases, with AC contributing 12.18%. The highest SCC incidence was in Sub-Saharan Africa (29.79 per 100,000 population). The AC incidence was highest in South-Eastern Asia (3.67 per 100,000 population). Age-specific trends showed SCC peaking at approximately age 55 years and AC plateauing after age 45 years.

**Conclusions:**

This study provided a comprehensive estimate of cervical cancer incidence by histological subtype. SCC remained the dominant subtype globally, whereas the incidence of AC varied across regions. These findings highlighted the need for tailored prevention strategies, especially testing for human papillomavirus to detect AC in high burden areas.

**Supplementary Information:**

The online version contains supplementary material available at 10.1007/s44197-023-00172-7.

## Background

Cervical cancer is the second most common cancer and the second most common cause of death from cancer among women of reproductive age worldwide [1]. Cervical cancer ranks the second among the most common lethal tumors in developing countries and ranks the tenth in developed countries, making this type of cancer an example of global inequality. In May 2018, the Director-General of the World Health Organization (WHO) announced a global call of action to eliminate cervical cancer as a public health problem. Strategies and roadmaps have been proposed to achieve this global goal. Vaccination against human papillomavirus (HPV), screening and treatment for precancerous lesions, and cancer management are the three pillars of cervical cancer prevention, and clear targets have been set to monitor the progress of cervical cancer elimination for the period 2020–2030 [2].

Cervical cancer has two main histological subtypes, namely, squamous cell carcinoma (SCC) and adenocarcinoma (AC). These two histological subtypes have distinct characteristics in terms of etiology, screening effectiveness, and prognosis. HPV infection is the main etiologic agent in both SCC and AC, although a greater proportion of AC is owing to infection with HPV 18, particularly in younger women [3]. Behavioral risk factors that can mediate the risk of HPV exposure also vary between SCC and AC [4, 5]. These risk factors include cigarette smoking, a known risk factor of SCC; however, the association of smoking with AC has been unclear and even a negative association has been reported [6]. The effectiveness of cervical cancer screening is distinguished according to histological subtype as well as screening methods. Papanicolaou screening (Pap smear) can help to detect precancerous lesions and early-stage SCC. However, AC lesions can be missed in Pap smear sampling owing to their location in the upper part of the endocervical canal. HPV DNA testing has been proven to be a useful tool for improving the detection of AC. The prognosis of AC and SCC also vary. AC has worse survival outcomes than SCC, as reported in patients with IA2–IIA2 cervical cancer [7]. A meta-analysis also revealed poorer outcomes of disease-free and overall survival in patients with AC undergoing definitive radiotherapy or concurrent chemoradiotherapy than in those with SCC undergoing similar treatments [8].

It is essential to estimate the global incidence of cervical cancer according to histological subtype to provide basic epidemiological data for this disease and to inform appropriate screening methods to accomplish the global goal of cervical elimination. However, previous epidemiological studies were focused on certain countries, and the global landscape of cervical cancer incidence according to histological subtype remained unclear. In this study, we aimed to estimate the global incidence of cervical cancer by histological subtype using high-quality population-based cancer registries to fill this research gap and to inform future cervical cancer prevention strategies.

## Methods

### Data Source

The Cancer Incidence in Five Continents (CI5) project was established in a collaboration between the International Agency for Research on Cancer and the International Association of Cancer Registries [9]. We obtained data of cervical cancer cases categorized by histological subtype from the CI5 Volumes XI, which were formed by 343 population-based cancer registries worldwide at the national or subnational level covering the period 2008‒2012. We obtained the estimated national number and rate of new cervical cancer cases in 2020 for 185 countries, by 5-year age groups and by geographic region, from the Global Cancer Observatory (GLOBOCAN) 2020 database [10, 11]. Histological subtypes of cervical cancer were defined according to the third edition of the International Classification of Diseases for Oncology as described in the CI5 volumes, as follows: SCC (8050–8078, 8083–8084) and AC (8140–8141, 8190–8211, 8230–8231, 8260–8265, 8310, 8380, 8382–8384, 8440–8490, 8570–8574, 8576) [12].

### Subtype Case Estimation

We estimated the number of cases of each cervical cancer subtype based on the ICD-O-3 codes, as described in the CI5 XI volumes. We excluded cancer registry data with a prevalence of cervical cancer with unspecified histology of ≥ 75% at the national level and fewer than one case of SCC and AC in each stratum. In total, 67 countries were included in the analysis, and national-level proportions of SCC, AC, other, and unspecified cases were calculated by 5-year age group. At regional level, the proportion of unspecified cases ranged from 4.84% of total cervical cancer cases in Oceania to 32.51% in Sub-Saharan Africa (Supplementary Table 1).

In the principal analysis, we assumed that unspecified cases would likely be SCC and AC and reallocated them to these subtypes as per their relative proportions. Country-specific subtype proportions were then applied to the incidence and number of new cervical cancer cases by 5-year age group, as estimated in GLOBOCAN 2020. For the remaining 113 countries in which country-specific cervical cancer registry data were unavailable or the exclusion criteria were met, the sub-regional average proportions of SCC, AC, and other specific histology were calculated by aggregating the reported number of cervical cancer cases in the individual countries. Subregions were classified using the United Nations definitions, as follows: the Caribbean, Central, America, Eastern Asia, Eastern Europe, North America, Northern Africa, Northern Europe, Oceania, South America, South-Central Asia, South-Eastern Asia, Southern Europe, Sub-Saharan Africa, Western Asia, and Western Europe [13]. Detailed information on the data sources for each country is provided in Supplementary Table 2.

Age-standardized rates (ASRs) of SCC, AC, and other specific histology per 100,000 women-years were calculated for each country and region in 2020 using the direct method and the World Standard Population [14]. Age trends of the crude rate of cervical cancer incidence were estimated separately by histological subtypes and by world regions.

### Sensitivity Analysis

In sensitivity analysis, we used an alternative scenario of reallocation in which cases with unspecified histology were allocated into the SCC, AC, and other specified histology groups in accordance with their relative proportions. Then, the incidence rate and number of cases of each cervical cancer subtypes were compared between the principal and sensitivity analyses.

## Results

**Fig. 1:**
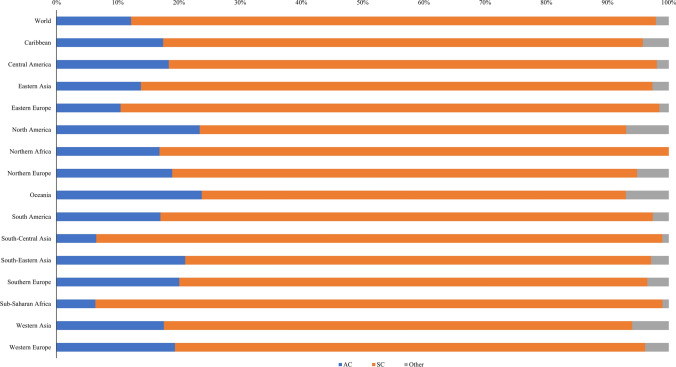
Proportion of cervical cancer cases by histological subtype in 2020, by geographic region. AC, adenocarcinoma; SCC, squamous cell carcinoma

SCC was the dominant histological subtype within the spectrum of cervical cancer, constituting 82.72% of the total reported cervical cancer cases on a global scale. Notably, SCC emerged as the principal histological subtype across diverse geographic regions, encompassing 15 distinct regions worldwide. This compositional prevalence spanned from 69.26% in Oceania to a very pronounced 92.63% in Sub-Saharan Africa. AC contributed to 12.18% of the overall incident cases of cervical cancer at global level, exhibiting variation in its distribution across regions. This proportion fluctuated from 6.35% in the Sub-Saharan African region to 23.72% in Oceania (Fig. 1).

**Fig. 2:**
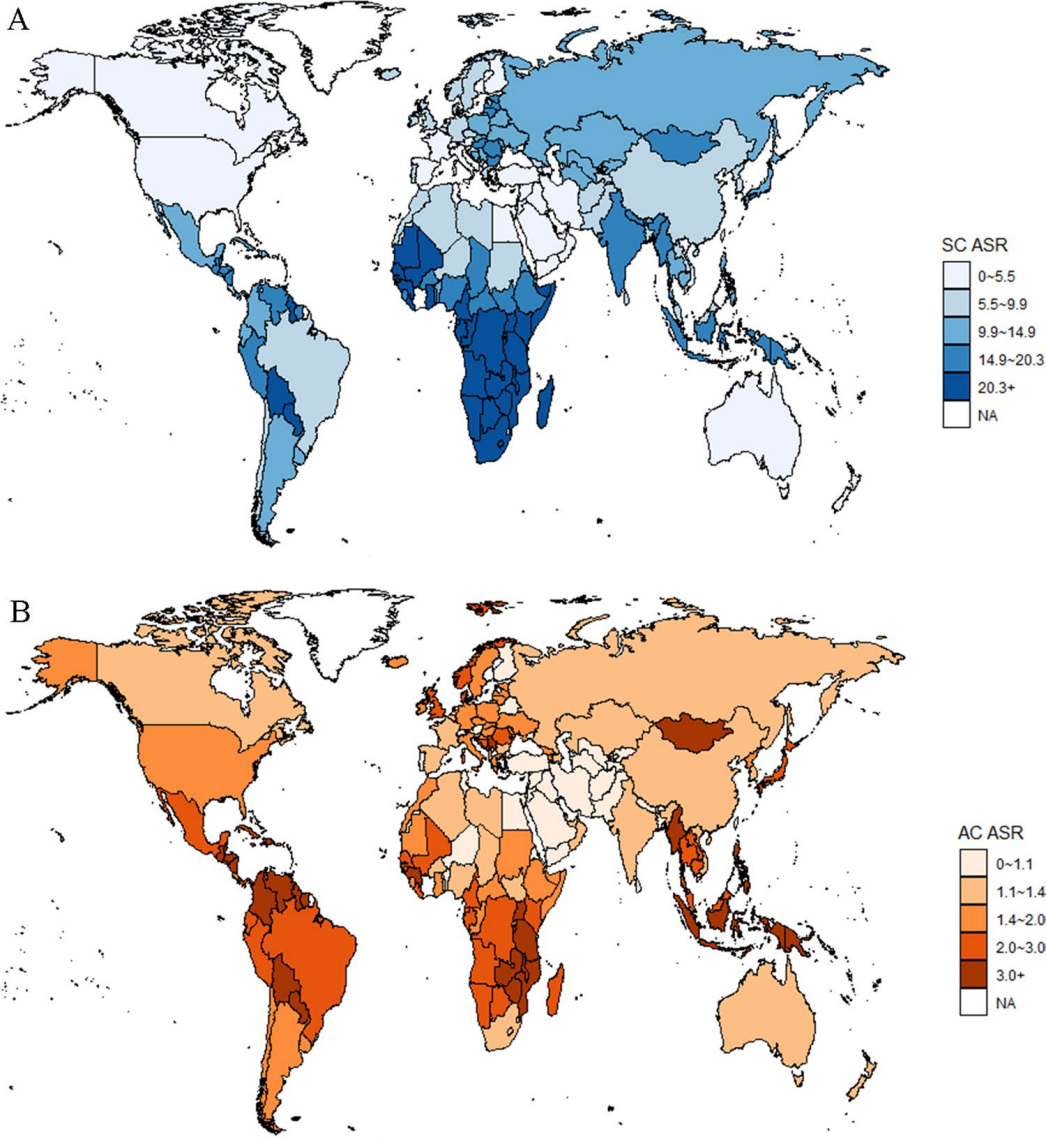
Age-standardized incidence rate (ASR) of cervical cancer by histological subtype per 100,000 women-years. **a** Squamous cell carcinoma (SCC); **b** Adenocarcinoma (AC)

Table 1 provided comprehensive details on the incidence of subtype-specific cervical cancer, incorporating both numerical counts and ASRs. Globally, SCC accounted for 507,303 cases, whereas a total of 72,070 AC cases were reported, corresponding to an ASR of 9.81 and 2.76 per 100,000 population, respectively. The subtype-specific cervical cancer burden varied across regions and countries. The highest incidence of SCC emerged as 29.79 per 100,000 population in Sub-Saharan Africa; the lowest incidence of SCC was in Western Asia with 3.15 per 100,000 population. For AC, the highest and lowest incidence was found in South-Eastern Asia (3.67 per 100,000 population) and Western Asia (0.77 per 100,000 population), respectively. In terms of the total number of cases, South-Central Asia (134,881 cases, 26.59% of global SCC cases) and Eastern Asia (17,390 cases, 24.13% of global AC cases) accounted for the largest number of incident cases of global cervical cancers in 2020. A global map of cervical cancer incidence by histological subtype was displayed in Fig. 2. The highest incidence rate of cervical SCC was in Malawi (63.07 per 100,000 population) and the lowest ASR of SCC was in Iraq (1.66 per 100,000 population). For cervical AC, the highest incidence rate emerged in Fiji (6.84 per 100,000 population) and the lowest was in the Islamic Republic of Iran (0.29 per 100,000 population).

**Table 1:** Estimated number of cases and age-standardized incidence rate of cervical cancer per 100,000 women-years by subtype and world region in 2020

Regions	AC		SCC		Other	
	Number of cases	ASR	Number of cases	ASR	Number of cases	ASR
World	72,070	2.76	507,303	9.81	12,470	0.75
Caribbean	612	2.48	2754	10.76	149	0.58
Central America	2445	2.57	10,633	10.99	261	0.27
Eastern Asia	17,390	1.73	105,471	8.67	3374	0.43
Eastern Europe	3322	1.52	27,944	12.67	492	0.26
North America	3409	1.46	10,149	4.26	1014	0.43
Northern Africa	1142	1.06	5654	5.15	0	0
Northern Europe	1214	1.99	4878	7.88	333	0.58
Oceania	569	2.4	1660	6.95	168	0.71
South America	6853	2.38	32,481	12.71	1053	0.31
South-Central Asia	9475	0.98	134,881	14.16	1561	0.16
South-Eastern Asia	14,188	3.67	51,346	13.6	1956	0.53
Southern Europe	1733	1.59	6611	5.85	303	0.26
Sub-Saharan Africa	6959	2.15	101,530	29.79	1125	0.36
Western Asia	922	0.77	4019	3.15	314	0.22
Western Europe	1837	1.38	7292	5.36	367	0.31

AC, adenocarcinoma; ASR, age-standardized rate; SCC, squamous cell carcinoma

**Fig. 3:**
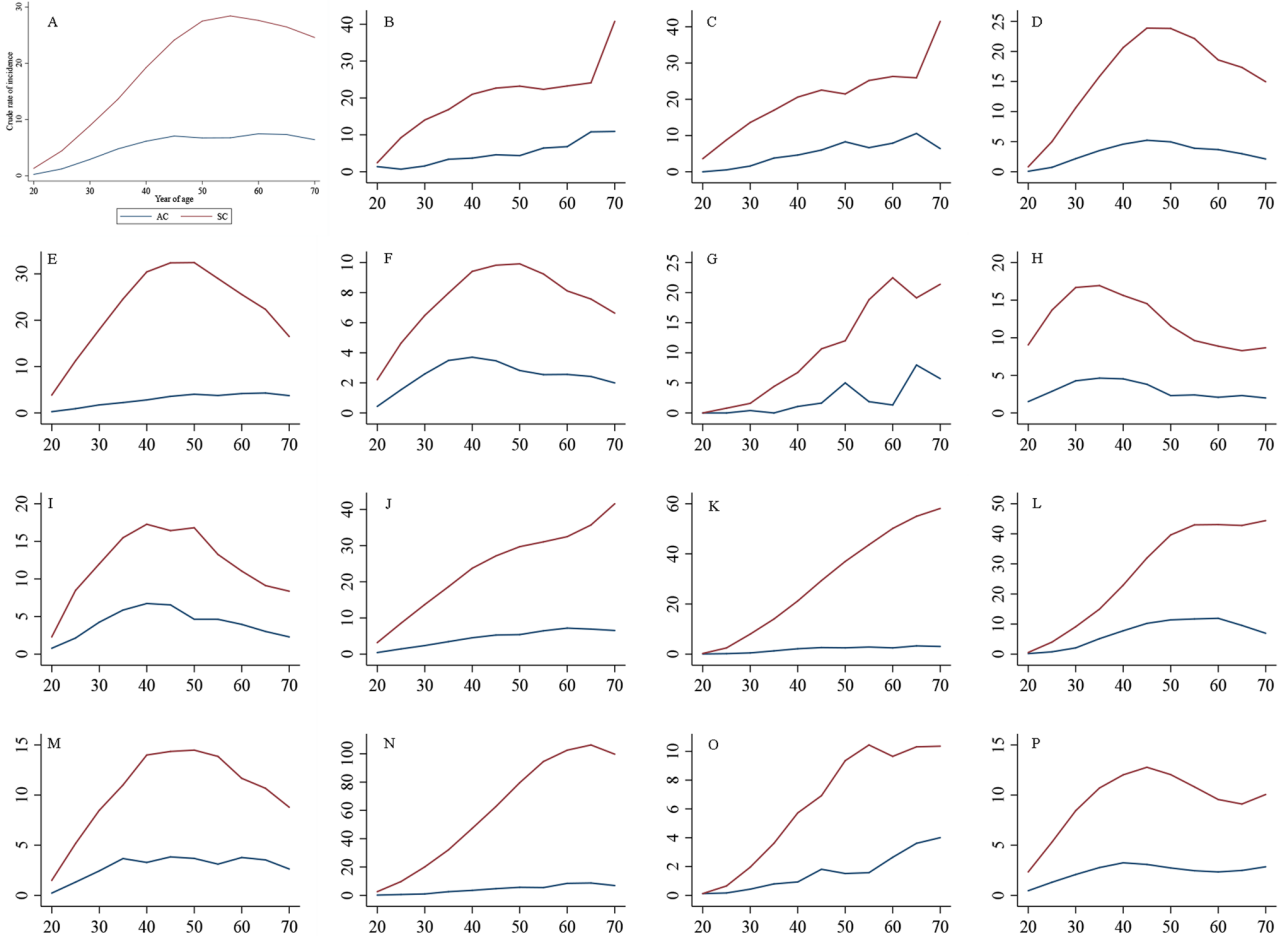
Age trend of cervical cancer incidence by histological subtype and by world region. **a** World; **b** Caribbean; **c** Central America; **d** Eastern Asia; **e** Central and Eastern Europe; **f** North America; **g** Northern Africa; **h** Northern Europe; **i** Oceania; **j** South America; **k** South-Central Asia; **l** South-Eastern Asia; **m** Southern Europe; **n** Sub-Saharan Africa; **o** Western Asia; **p** Western Europe

Figure 3 showed the trajectory of cervical cancer subtypes with increasing age. On a global scale, the incidence of cervical SCC displayed an increasing trend with age, and peaked at approximately 55 years of age, and subsequently undergoing a gradual descent. In contrast, the age-specific incidence of AC exhibited a gradual ascent from ages 20–45 years, with a subsequent plateau after age 45 years. At regional level, age trends in SCC incidence showed different patterns across regions. In Eastern Asia, Central and Eastern Europe, North America, Northern Europe, Oceania, Southern Europe, and Western Europe, the age-specific incidence of SCC followed an inverted U-shape. Alternatively, the Caribbean, Central America, Northern Africa, South America, South-Central Asia, South-Eastern Asia, Sub-Saharan Africa, and Western Asia demonstrated an upward trajectory of cervical SCC incidence with increasing age.

In the alternative scenario, we reallocated unspecified histology cases into the SCC, AC, and other specified histology groups as per their relative proportions. The proportion and disease burden of the main histological subtypes were presented in Supplementary Table 3 and Supplementary Figure 1. The robust results illustrated that the proportions of SCC, AC, and other specified histological subtypes were 81.41%, 11.35%, and 7.24%, respectively. The estimated number of cases was 481,807 for SCC (ASR = 9.76 per 100,000 women-years), 67,205 for AC (ASR = 2.75 per 100,000 women-years) and 42,847 for other specified histology (ASR = 0.82 per 100,000 women-years).

## Discussion

Cervical cancer is a global health concern and holds the distinction of being the first noncommunicable disease with a global commitment for elimination. Cervical cancer comprises two main histological subtypes, namely, SCC and AC. Estimation based on global population-based registry data in the current study revealed that SCC is the dominant subtype, and the incidence of subtype-specific cervical cancer varies across geographic regions and age groups. Estimation of the histology-specific burden within cervical carcinoma is of importance for the formulation of tailored prevention strategies to achieve the overarching goal of global cervical cancer elimination.

In 2020, WHO adopted a global strategy to reduce the incidence of cervical cancer to the threshold of four cases per 100,000 women within the twenty-first century [2]. In pursuit of this ambitious goal, understanding of the histological subtypes is important to shape strategies and approaches. The effectiveness of cervical screening exhibits divergent profiles across histologic subtypes. Pap smear has greater sensitivity in detecting precursor lesions of SCC; however, diagnostic discernment using Pap smear for precursor lesions in cervical AC is comparatively poor. Implementation of human papillomavirus testing as a primary screening method represents a prospective avenue toward reducing the incidence of invasive AC. Consequently, as a crucial public health investment, the selection of technologies and methods for cervical cancer screening should be determined in response to the subtype-specific incidence.

We found that SCC was the most common histological subtype of cervical cancer. Similar proportions of each cervical cancer histological subtype have been reported based on country-specific data. A nationwide study in the United States found that the incidence of SCC surpassed that of AC in all age groups [15]. The subtype-specific cervical cancer incidence in Canada suggested a parallel dominant trend [16]. The present study also revealed regional disparity in the distribution of histological subtypes in that over 90% of the total cervical cancer cases were identified as SCC in Sub-Saharan Africa and South-Central Asia, whereas this proportion was less than 60% in Oceania and North America. This difference in the distribution across regions by histological subtype may be a result of the complex interaction between cervical cancer carcinogenesis and prevention strategies. Cervical cancer screening strategies, rooted in cytology-based methods, have been featured in guidelines and practice since the 1960s, most notably in the United States and Europe. Owing to the superior sensitivity of Pap testing in detecting SCC and its precursors, a discernible downward trend in cervical cancer incidence has been observed which was catalyzed by the decrease in SCC incidence [17, 18]. Consequently, regions with sustained cervical cancer screening efforts, such as the United States and Europe, have witnessed a comparatively lower prevalence of SCC, in contrast to regions that have lacked similar screening initiatives, typified by the case of Sub-Saharan Africa. SCC incidence in this region surged to 29.79 per 100,000 population, exceeding threefold of the global average level. This regional disparity highlighted the benefit of cervical cancer screening and spotlighted Sub-Saharan Africa as a focus in global cervical cancer prevention.

Cervical AC merits greater attention in the formulation of strategies for cervical cancer prevention. Globally, approximately one-eighth of cervical cancer cases were AC, with this proportion reaching approximately one-fourth in Oceania and North America. Countries in North America and Europe have observed a continuously increasing trend of AC in the past decades [19–23]. However, the reasons for this trend cannot be fully explained by a change in sexual behaviors. Thus, it is imperative to underscore preventive strategies with high efficacy in mitigating the incidence of AC and their integration within both clinical protocols and preventive guidelines. The findings of the present investigation advocated adoption of HPV testing as the primary cervical cancer screening method across Oceania, North America, and Europe. Previous observational studies [24] and randomized controlled trials [25] have reported a greater sensitivity of DNA testing than cytologic testing for detection of cervical intraepithelial neoplasia (CIN). Seven prospective HPV studies reported that the cumulative incidence rate of CIN grade 3 or cancer after 6 years was considerably lower among women who were negative for HPV at baseline (0.27%) than among those with negative results on cytology (0.97%) [26]. DNA testing has been found to beof higher effectiveness in preventing AC than cytology-based testing. A pooled analysis of four European randomized trials confirmed a larger gain for AC than for SCC with HPV testing as the primary screening strategy [27]. Consequently, the heightened prevalence of cervical AC in Oceania, North America, and Northern Europe highlighted the need to reevaluate the primary screening testing in these regions.

This study has several strengths. We estimated the subtype-specific cervical cancer burden based on high-quality registry data from the CI5 platform. The results can inform cervical cancer prevention strategies to achieve the global elimination goal. This study also has some limitations. With the greater implementation of cervical screening programs, a greater proportion of early stage cervical cancer cases should be identified. However, in this study, the cervical cancer burden by stage at diagnosis could not be estimated owing to data unavailability.

## Conclusions

Estimation of the cervical cancer incidence by histological subtype revealed that SCC remains the dominant subtype, especially in Sub-Saharan Africa and South-Central Asia. AC prevention should be emphasized especially in Oceania, North America, and Northern Europe. Tailored cervical cancer screening strategies are warranted according to histology-based incidence estimation to achieve the global goal of cervical cancer elimination.

### Supplementary Information

Below is the link to the electronic supplementary material.

Supplementary file1 (DOCX 889 KB)

## Data Availability

The data sets generated and/or analyzed during the current study are available in the Cancer Incidence in Five Continents project (https://gco.iarc.fr/ci5#ci5-XI) and Global Cancer Observatory 2020 database (https://gco.iarc.fr/).

## Abbreviations

AC: Adenocarcinoma
ASR: Age-standardized rate
CI5: Cancer Incidence in Five Continents
GLOBOCAN: Global Cancer Observatory
HPV: Human papillomavirus
Pap: Papanicolaou
SCC: Squamous cell carcinoma
WHO: World Health Organization
