# Supplementary material for: Global Cervical Cancer Incidence by Histological Subtype and Implications for Screening Methods
Source: J Epidemiol Glob Health. 2024 Jan 3;14(1):94–101. doi: 10.1007/s44197-023-00172-7 (PMC11043316; doi:10.1007/s44197-023-00172-7)
Supplement: Supplementary file 1 — Supplementary file1 (DOCX 889 KB) [file 44197_2023_172_MOESM1_ESM.docx]

**Supplemental Materials**

**Global Cervical Cancer Incidence by Histological Subtype and Implications for Screening Methods**

Minmin Wang, Ph.D., Kepei Huang, B.S., Jia Wang, Ph.D., Yinzi Jin, Ph.D., Zhi-Jie Zheng, M.D., Ph.D.

Supplemental Table 1. Distribution of cervical cancer subtypes as a proportion of total lung cancer cases, before reallocation of unspecified cases: adenocarcinoma (AC), squamous cell carcinoma (SC), other specified histology, and unspecified according to world region.

Supplemental Table 2. Method used for country estimates: country-specific or regional average.

Supplemental Table 3. Estimated number of cases and age-standardized incidence rate (ASR) of cervical cancer per 100,000 person-years by subtype and world region in 2020 according to sensitivity analysis: unspecified cases reallocated to adenocarcinoma (AC), squamous cell carcinoma (SC), and other specified histology.

Supplemental Figure 1. Age-standardized incidence rate (ASR) of cervical cancer by histological subtype per 100,000 person-years in sensitivity analysis. (a) Squamous cell carcinoma (SC); (b) adenocarcinoma (AC).

**Supplemental Table 1. Distribution of cervical cancer subtypes as a proportion of total lung cancer cases, before reallocation of unspecified cases: adenocarcinoma (AC), squamous cell carcinoma (SC), other specified histology, and unspecified according to world region.**

| **Region** | **AC** | **SC** | **Other** | **Unspecified histology** |
| --- | --- | --- | --- | --- |
| **Caribbean** | 15.99 | 73 | 4.09 | 6.91 |
| **Central America** | 12.62 | 58.18 | 1.87 | 27.34 |
| **Eastern Asia** | 14.06 | 72.2 | 3.76 | 9.99 |
| **Eastern Europe** | 10.63 | 80.41 | 1.88 | 7.08 |
| **North America** | 22.02 | 64.64 | 6.94 | 6.4 |
| **Northern Africa** | 15.13 | 68.49 | 0 | 16.39 |
| **Northern Europe** | 17.22 | 69.51 | 4.57 | 8.71 |
| **Oceania** | 22.23 | 65.89 | 7.05 | 4.84 |
| **South America** | 13.56 | 72.59 | 2 | 11.85 |
| **South-Central Asia** | 5.3 | 74.22 | 1.09 | 19.38 |
| **South-Eastern Asia** | 16.78 | 60.79 | 2.98 | 19.46 |
| **Southern Europe** | 18.95 | 68.03 | 3.41 | 9.61 |
| **Sub-Saharan Africa** | 4.43 | 62.11 | 0.96 | 32.51 |
| **Western Asia** | 16.41 | 69.47 | 5.31 | 8.81 |
| **Western Europe** | 17.72 | 69.62 | 3.93 | 8.73 |

**Supplemental Table 2. Method used for country estimates: country-specific or regional average.**

| **Country** | **Methods** |
| --- | --- |
| Bahamas | Region average |
| Barbados | Region average |
| Cuba | Region average |
| Dominican Republic | Region average |
| France, Martinique | Country-specific |
| Haiti | Region average |
| Jamaica | Country-specific |
| Puerto Rico | Country-specific |
| Saint Lucia | Region average |
| Trinidad and Tobago | Region average |
| Belize | Region average |
| Costa Rica | Country-specific |
| El Salvador | Region average |
| Guatemala | Region average |
| Honduras | Region average |
| Mexico | Region average |
| Nicaragua | Region average |
| Panama | Region average |
| China | Country-specific |
| Japan | Country-specific |
| Korea, Democratic Republic of | Region average |
| Korea, Republic of | Country-specific |
| Mongolia | Region average |
| Belarus | Country-specific |
| Bulgaria | Country-specific |
| Czechia | Country-specific |
| Hungary | Region average |
| Poland | Country-specific |
| Republic of Moldova | Region average |
| Romania | Region average |
| Russian Federation | Country-specific |
| Slovakia | Country-specific |
| Ukraine | Country-specific |
| Canada | Country-specific |
| United States of America | Country-specific |
| Algeria | Country-specific |
| Egypt | Region average |
| Libya | Region average |
| Morocco | Region average |
| Sudan | Region average |
| Tunisia | Region average |
| Denmark | Country-specific |
| Estonia | Country-specific |
| Finland | Region average |
| Iceland | Country-specific |
| Ireland | Country-specific |
| Latvia | Country-specific |
| Lithuania | Country-specific |
| Norway | Country-specific |
| Sweden | Region average |
| United Kingdom | Country-specific |
| Australia | Country-specific |
| Fiji | Region average |
| France, New Caledonia | Country-specific |
| New Zealand | Country-specific |
| Papua New Guinea | Region average |
| Samoa | Region average |
| Solomon Islands | Region average |
| Vanuatu | Region average |
| Argentina | Country-specific |
| Bolivia, Plurinational State of | Region average |
| Brazil | Country-specific |
| Chile | Country-specific |
| Colombia | Country-specific |
| Ecuador | Country-specific |
| French Guiana | Country-specific |
| Guyana | Region average |
| Paraguay | Region average |
| Peru | Country-specific |
| Suriname | Region average |
| Uruguay | Country-specific |
| Venezuela, Bolivarian Republic of | Region average |
| Afghanistan | Region average |
| Bangladesh | Region average |
| Bhutan | Region average |
| India | Country-specific |
| Iran, Islamic Republic of | Country-specific |
| Kazakhstan | Region average |
| Kyrgyzstan | Region average |
| Maldives | Region average |
| Nepal | Region average |
| Pakistan | Region average |
| Sri Lanka | Region average |
| Tajikistan | Region average |
| Turkmenistan | Region average |
| Uzbekistan | Region average |
| Brunei Darussalam | Country-specific |
| Cambodia | Region average |
| Indonesia | Region average |
| Lao People's Democratic Republic | Region average |
| Malaysia | Country-specific |
| Myanmar | Region average |
| Philippines | Country-specific |
| Singapore | Region average |
| Thailand | Country-specific |
| Timor-Leste | Region average |
| Viet Nam | Country-specific |
| Albania | Region average |
| Bosnia and Herzegovina | Region average |
| Croatia | Country-specific |
| Greece | Region average |
| Italy | Country-specific |
| Malta | Country-specific |
| Montenegro | Region average |
| North Macedonia | Region average |
| Portugal | Country-specific |
| Serbia | Region average |
| Slovenia | Country-specific |
| Spain | Country-specific |
| Armenia | Region average |
| Azerbaijan | Region average |
| Bahrain | Country-specific |
| Cyprus | Country-specific |
| Georgia | Region average |
| Iraq | Region average |
| Israel | Country-specific |
| Jordan | Country-specific |
| Kuwait | Country-specific |
| Lebanon | Region average |
| Oman | Region average |
| Qatar | Country-specific |
| Saudi Arabia | Country-specific |
| Syrian Arab Republic | Region average |
| Turkey | Country-specific |
| United Arab Emirates | Region average |
| Yemen | Region average |
| Austria | Country-specific |
| Belgium | Country-specific |
| France | Country-specific |
| Germany | Country-specific |
| Luxembourg | Region average |
| Switzerland | Country-specific |
| The Netherlands | Country-specific |
| Angola | Region average |
| Benin | Region average |
| Botswana | Region average |
| Burkina Faso | Region average |
| Burundi | Region average |
| Cabo Verde | Region average |
| Cameroon | Region average |
| Central African Republic | Region average |
| Chad | Region average |
| Comoros | Region average |
| Congo, Democratic Republic of | Region average |
| Congo, Republic of | Region average |
| Côte d'Ivoire | Region average |
| Djibouti | Region average |
| Equatorial Guinea | Region average |
| Eritrea | Region average |
| Eswatini | Region average |
| Ethiopia | Region average |
| Gabon | Region average |
| Ghana | Region average |
| Guinea | Region average |
| Guinea-Bissau | Region average |
| Kenya | Country-specific |
| Lesotho | Region average |
| Liberia | Region average |
| Madagascar | Region average |
| Malawi | Region average |
| Mali | Region average |
| Mauritania | Region average |
| Mauritius | Region average |
| Mozambique | Region average |
| Namibia | Region average |
| Niger | Region average |
| Nigeria | Region average |
| Rwanda | Region average |
| Sao Tome and Principe | Region average |
| Senegal | Region average |
| Sierra Leone | Region average |
| Somalia | Region average |
| South Africa | Country-specific |
| South Sudan | Region average |
| Tanzania, United Republic of | Region average |
| The Republic of the Gambia | Region average |
| Togo | Region average |
| Uganda | Country-specific |
| Zambia | Region average |
| Zimbabwe | Country-specific |

**Supplemental Table 3. Estimated number of cases and age-standardized incidence rate (ASR) of cervical cancer per 100,000 person-years by subtype and world region in 2020 according to sensitivity analysis: unspecified cases reallocated to adenocarcinoma (AC), squamous cell carcinoma (SC), and other specified histology.**

| **Region** | **AC** | | **SC** | | **Other** | |
| --- | --- | --- | --- | --- | --- | --- |
|  | Number of cases | ASR | Number of cases | ASR | Number of cases | ASR |
| **World** | 67205 | 2.75 | 481807 | 9.76 | 42847 | 0.82 |
| **Caribbean** | 601 | 2.47 | 2723 | 10.72 | 191 | 0.63 |
| **Central America** | 2420 | 2.55 | 10533 | 10.91 | 386 | 0.37 |
| **Eastern Asia** | 15861 | 1.72 | 96804 | 8.63 | 13586 | 0.47 |
| **Eastern Europe** | 3086 | 1.52 | 26228 | 12.65 | 2444 | 0.28 |
| **North America** | 2947 | 1.45 | 8813 | 4.24 | 2812 | 0.46 |
| **Northern Africa** | 1101 | 1.06 | 5505 | 5.15 | 190 | 0 |
| **Northern Europe** | 1086 | 1.98 | 4413 | 7.85 | 926 | 0.62 |
| **Oceania** | 531 | 2.4 | 1555 | 6.93 | 311 | 0.74 |
| **South America** | 6136 | 2.37 | 29427 | 12.68 | 4823 | 0.36 |
| **South-Central Asia** | 9100 | 0.98 | 130117 | 14.12 | 6702 | 0.2 |
| **South-Eastern Asia** | 13464 | 3.64 | 48994 | 13.5 | 5031 | 0.65 |
| **Southern Europe** | 1552 | 1.58 | 6039 | 5.83 | 1057 | 0.29 |
| **Sub-Saharan Africa** | 6861 | 2.14 | 100451 | 29.63 | 2302 | 0.52 |
| **Western Asia** | 858 | 0.77 | 3811 | 3.13 | 585 | 0.24 |
| **Western Europe** | 1601 | 1.37 | 6395 | 5.35 | 1501 | 0.33 |


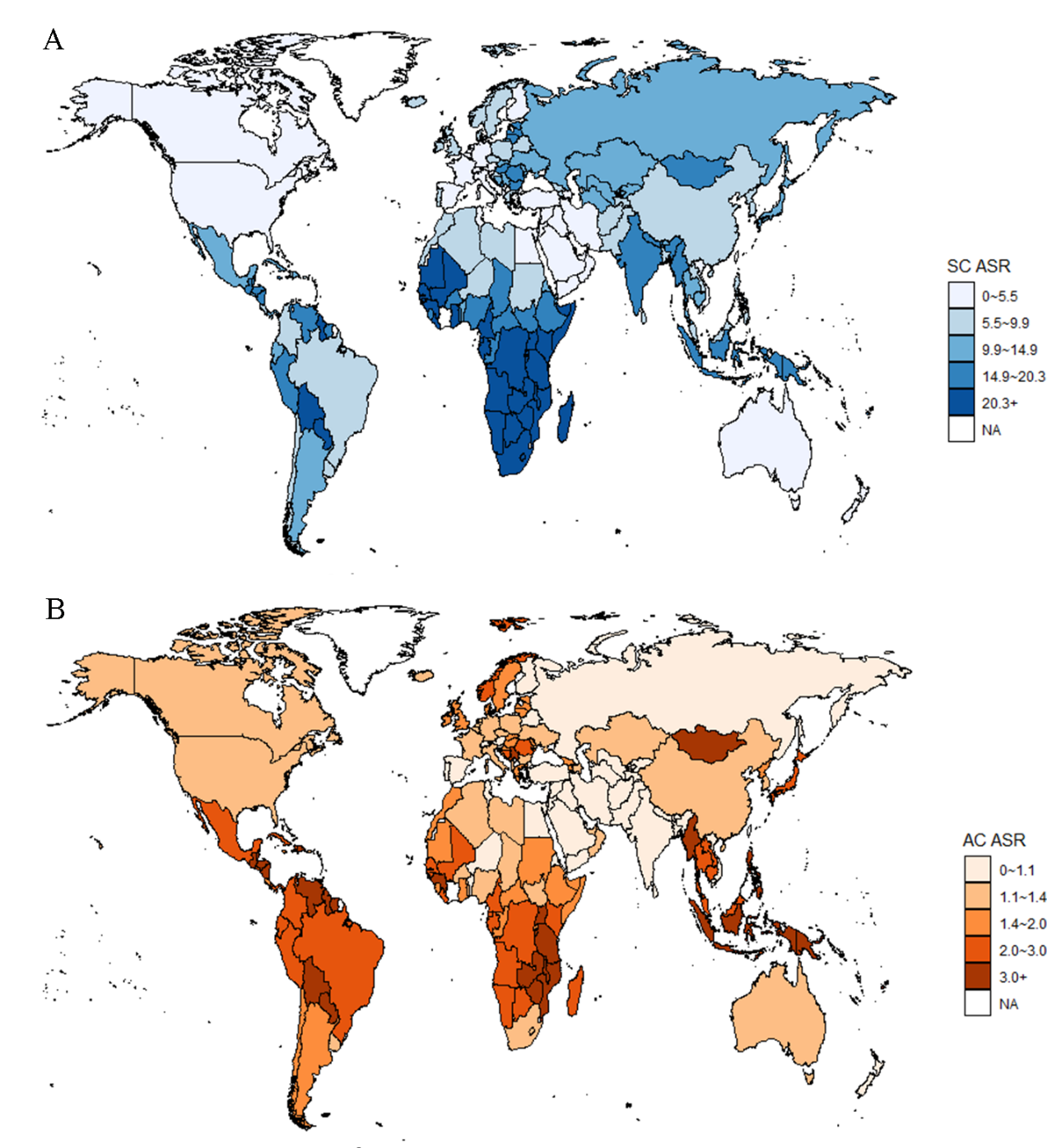


**Supplemental Figure 1. Age-standardized incidence rate (ASR) of cervical cancer by histological subtype per 100,000 person-years in sensitivity analysis. (a) Squamous cell carcinoma (SC); (b) adenocarcinoma (AC).**
